# Association of Visiting the Physiotherapist with Mortality in the Spanish General Population: A Population-Based Cohort Study

**DOI:** 10.3390/medicina59122187

**Published:** 2023-12-16

**Authors:** Rauf Nouni-García, Álvaro Carbonell-Soliva, Domingo Orozco-Beltrán, Adriana López-Pineda, María Isabel Tomás-Rodríguez, Vicente F. Gil-Guillén, José A. Quesada, Concepción Carratalá-Munuera

**Affiliations:** 1 Institute of Health and Biomedical Research of Alicante, General University Hospital of Alicante, Diagnostic Center, Fifth Floor, Pintor Baeza Street, 12, 03110 Alicante, Spain; raufrng@gmail.com (R.N.-G.); vte.gil@gmail.com (V.F.G.-G.); 2 Network for Research on Chronicity, Primary Care and Health Promotion (RICAPPS), 03550 San Juan de Alicante, Spain; drorozco@umh.es (D.O.-B.); jquesada@umh.es (J.A.Q.); maria.carratala@umh.es (C.C.-M.); 3 Clinical Medicine Department, School of Medicine, University of Miguel Hernández de Elche, Ctra, Nacional N-332 s/n, 03550 San Juan de Alicante, Spain; alvarocs108@gmail.com; 4 The Foundation for the Promotion of Health and Biomedical Research of Valencia Region (FISABIO), University Hospital of San Juan de Alicante, Ctra, Nacional N-332 s/n, 03550 San Juan de Alicante, Spain; 5 Pathology and Surgery Department, School of Medicine, University of Miguel Hernández de Elche, Ctra, Nacional N-332 s/n, 03550 Alicante, Spain; mitomas@umh.es

**Keywords:** physiotherapy, mortality, prevention

## Abstract

*Background and Objectives*: The purpose of this retrospective population-based cohort study was to analyse the association between attendance of physiotherapy with mortality in the Spanish general population and describe the profile of people who do not visit a physiotherapist in Spain. *Material and Methods*: The data sources were the 2011/2012 National Health Survey (ENSE11) and the national database of death in Spain, and the participants were all adult respondents in the ENSE11. *Results*: Of 20,397 people, 1101 (5.4%) visited the physiotherapist the previous year, and the cumulative incidence of total mortality was 5.4% (n = 1107) at a mean follow-up of 6.2 years. Visiting the physiotherapist was associated with lower all-cause mortality in the population residing in Spain, quantified at 30.1% [RR = 0.699; 95% CI (0.528–0.927); *p* = 0.013]. The factors associated with not visiting a physiotherapist were the following: rating one’s health as good (9.8%; n = 1017; *p* < 0.001), not having any hospital admission in the previous year (9.6%; n = 1788; *p* < 0.001), not having visited the general practitioner in the previous month (9.6%; n = 1408; *p* < 0.001), and not having attended a day hospital in the previous year (9.7%; n = 1836; *p* < 0.001). *Conclusions*: Visiting a physiotherapist was associated with a lower mortality from all causes in the population living in Spain.

## 1. Introduction

The World Confederation of Physiotherapy (WCPT) [1] considers that physiotherapists are trained to meet the functional needs of people with disabilities. They also play a vital role in preventing and reducing health problems associated with disability. In and of itself, according to the American Physical Therapy Association (APTA), physiotherapy is a profession directed to the care of the individual, their family, and their community and focuses on the study and analysis of human body movement in order to improve the individual’s quality of life and contribute to their social development [2]. In this way, physiotherapists, in addition to their role in secondary and tertiary interventions, also participate in the identification of risks and behaviours that prevent or hinder the optimal development of human movement. In addition, they also take part in health promotion interventions, enabling people to have greater control over the determinants of health [3].

Regarding the functions of a physiotherapist, in Spain, the assistance function is the most important, with physiotherapists forming part of a multidisciplinary team. In general, the assistance function consists of the direct relationship that a physiotherapist maintains with a healthy/sick individual or society in order to prevent, cure, and recover injuries through professional actions, consisting of establishing, applying, and evaluating the methods, actions, and techniques of physiotherapy. A physiotherapist establishes a relationship with a healthy or sick individual in the psychological, communicative, and physical dimensions via physical means [4]. The Spanish public health system has just over 5000 physiotherapists, a ratio of 0.1 per 1000 inhabitants, far below the recommendations of the World Health Organization (WHO) of 1 physiotherapist per 1000 inhabitants [5]. Approximately 90% of Spanish physiotherapists work in the private sector, representing a ratio of 0.9/1000 inhabitants [6]. The Spanish public physiotherapy service has long waiting lists, and private sector interventions are not refunded by the public system. In terms of access to physiotherapy services through public health systems and the co-payment or lack thereof of said services, these vary widely in Europe [7].

According to a Global Burden of Disease Study in 2019 [8], musculoskeletal disorders ranked first in terms of years lived with disability. Physiotherapists have an important role in treating and preventing disability [1]. The prevalence of musculoskeletal disorders is very high in the elderly, being an important cause of pain, which can influence mood, physical functioning, physical inactivity, and social interaction and be a cause of a sedentary lifestyle [9]. Promotion of physical activity among the elderly plays a key role in healthy aging and may also have an impact beyond functionality, influencing mental health, quality of life, and well-being [10,11,12]. Previous evidence shows that subjective well-being is associated with reduced risk of all-cause mortality and incidence of specific conditions [13]. Physiotherapy can help society in the prevention and treatment of activity limitations and/or participation restrictions [12] in people at risk of movement disorders that, if not adequately prevented or treated, could lead to reduced physical activity, a lower quality of life, and a more sedentary lifestyle, which are associated with certain types of diseases and, consequently, an increased risk of mortality [9].

To the best of our knowledge, in Spain, no study has previously analysed the association between physiotherapy care and mortality in the general population. However, previous studies have associated certain physiotherapy interventions with a decrease in the risk factors associated with higher mortality [10,13,14,15]. The aim of this study was to analyse the association between attendance of physiotherapy and mortality in the general population from all causes after six years and describe the profile of patients who do not visit a physiotherapist in Spain, using the 2011/2012 National Health Survey.

## 2. Materials and Methods

### 2.1. Study Design

This was a population-based retrospective linkage cohort study. The baseline variables were collected from the Spanish National Health Survey 2011/2012 (ENSE11) [16] data source. The response variable was mortality from any cause (yes/no), obtained by crossing the ENSE11 database and the national database of deaths ordered by cause of death. This linkage was carried out by the National Institute of Statistics (INE) [17] through a probabilistic cross-link based on name, surname, date of birth, sex, personal identification number, and province of birth, using the Levenshtein distance metric, with a linkage quality higher than 97%. The linkage was carried out between the years 2011 and 2017, establishing the duration of follow-up to measure mortality between the 1 July 2011 and the 31 December 2017, for a total follow-up period of 6.4 years.

### 2.2. Participants

The period of inclusion for the ENSE11 respondents was between July 2011 and June 2012, covering a total of 21,007 adults (15 years and over). The ENSE11 has a national geographic scope and used a stratified three-stage sampling design. The units of the first stage were the census sections (population units of about 5000 inhabitants), the second-stage units were family dwellings, and, within each household, the third-stage units were adults aged 15 or over. A different sample was designed for each of the 17 regions of Spain, and was stratified by the size of the municipality. The survey represents all adults residing in Spain in 2011, about 39.7 million people. The present study included all ENSE11 adult participants and excluded those for whom information on the study variables was not available.

### 2.3. Variables/Outcomes Measures

The outcome variable was mortality from any cause (yes/no). The exposure variable was to answer question 52 of the ENSE11: “During the previous 12 months, have you visited a physiotherapist for yourself? (yes/no)”. The explanatory variables, which were considered potentially confounding, included five types:

- Socio-demographic variables: sex, age group, autonomous community of residence, size of municipality, social working class [18], body mass index, country of birth, marital status, educational level, and net monthly household income.
- Lifestyle habits: dental hygiene, tobacco use, exposure to tobacco smoke, risk of alcohol consumption, sleeping hours, main daily activity, and main leisure activity.
- Diet: breakfast, consumption of fruit, vegetables, legumes, dairy products, cakes and pastries, and fast food.
- Health and comorbidities: self-perceived health, presence of any chronic disease, high blood pressure (HBP), acute myocardial infarction (AMI), other heart disease, varicose veins in the legs, osteoarthritis, arthritis or rheumatism, chronic cervical pain, chronic low back pain, chronic allergy, asthma, chronic bronchitis, emphysema or chronic obstructive pulmonary disease (COPD); diabetes, stomach ulcer or duodenum, urinary incontinence, high cholesterol, cataracts, chronic skin problems, chronic constipation; cirrhosis or liver dysfunction; chronic depression; chronic anxiety; other mental problems; embolism, cerebral infarction or cerebral haemorrhage (stroke); migraine or frequent headache; haemorrhoids; malignant tumours; osteoporosis; thyroid problems; permanent injuries or defects caused by an accident or accidents in the previous year; mental health (GHQ12); use of glasses or contact lenses; use of hearing aids; flu vaccine in the last campaign; activity restriction (limitation of activity for health reasons in the previous two weeks); being bedridden for medical reasons in the previous two weeks; and limitation of daily activity in the previous six months.
- Use of health services: hospital admission in the previous year; visit to the primary care doctor in the previous month; visit to the specialist doctor in the previous month; visit to the day hospital in the previous year; visit to the psychologist in the previous year; and diagnostic tests performed in the previous year (X-ray; computed tomography scan (CT); ultrasound; nuclear magnetic resonance (MRN)).

Table S1 of the additional materials details the code of the survey question that collects the information for each variable as well as the answer options. All information collected through the survey is self-reported by the responder at the time of the survey.

### 2.4. Statistical Analysis

A descriptive analysis of all the variables was carried out by calculating frequencies for the qualitative variables. The factors associated with the visit to the physiotherapist and the incidence of mortality were analysed using contingency tables, applying the chi-squared test. As crude measures of association, we calculated the cumulative incidence (CI) of mortality in the exposed group (Ie) and the incidence in the unexposed group (Iu), the unadjusted relative risk (RRc) value as the ratio of cumulative incidence (RRc = Ie/Iu), absolute risk reduction (ARR) as the difference in incidence (ARR = Iu − Ie), relative risk reduction (RRR) as complementary to RR (RRR = 1 − RR), and exposure impact number (EIN) as the inverse of ARR (EIN = 1/ARR), interpreted as the number of individuals with exposure among whom one excess case is due to the exposure [19].

To estimate the magnitudes of associations with incidence of mortality, adjusted relative risks (RRa) have been estimated together with their 95% confidence intervals, by adjusting multivariate Poisson models with robust variance [20]. A stepwise variable selection procedure was carried out based on the Akaike information criterion (AIC), and the possible confusion of the explanatory variables by the effect of visiting the physiotherapist on the incidence of mortality was evaluated. The possible multicollinearity of the variables was also taken into account. A model validation process was carried out, dividing the sample randomly into two subsamples: training to adjust the model (70% of the total) and testing to validate it (30% of the total), obtaining an honest estimate of the predictive capacity of the model using the area under the ROC curve and its 95% confidence interval in the testing sample. To obtain representative estimates of the Spanish population, complex sampling was taken into account, using the survey elevation factor divided by its mean as a weighting factor, obtaining weights centred on its mean [21]. The analyses were performed using SPSS v.28 [22] and R v.4.0.2 software [23].

## 3. Results

**Figure 1:**
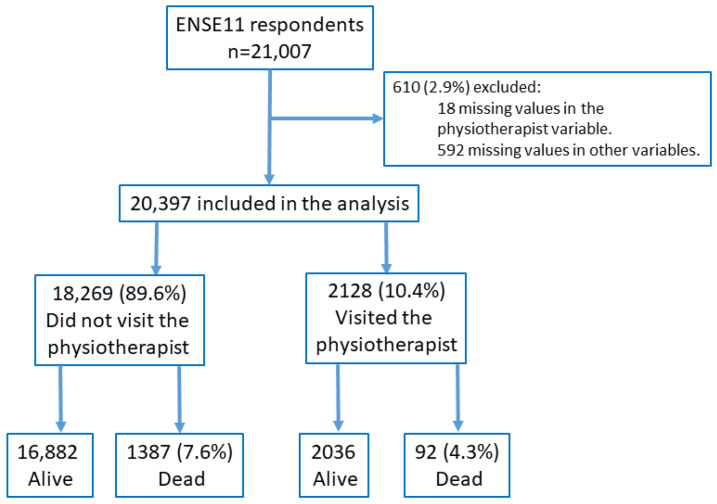
Flow chart of the study.

Of the total of 21,007 adults (15 years or older) surveyed, 18 (0.08%) participants were excluded for not presenting values in the exposure variable (visit to the physiotherapist) and 592 (2.8%) subjects for not presenting valid values in the rest of the explanatory variables, obtaining a total of 20,397 participants for analysis. Figure 1 shows the study flow chart.

Of the 20,397 participants included in this study, 5.4% (n = 1101) visited the physiotherapist the previous year, 51.3% (n = 10,455) were women, 85.7% (n = 17,470) were born in Spain, 15.4% (n = 3144) had obesity, 33.6% (n = 6848) had secondary education, 45.1% (n = 9199) reported standing most of the day, 44.4% (n = 9051) did not perform any physical leisure activity, 51.0% (n = 10,413) rated their self-perceived health as good, 18.3% (n = 3734) had osteoarthritis, 27.3% (n = 5566) had an X-ray in the previous year, and 7.4% (n = 1513) had an MRI during the previous year. The description of the study sample according to the study variables is presented in Table 1 in summary form (Table S2 in extended form).

**Figure 2:**
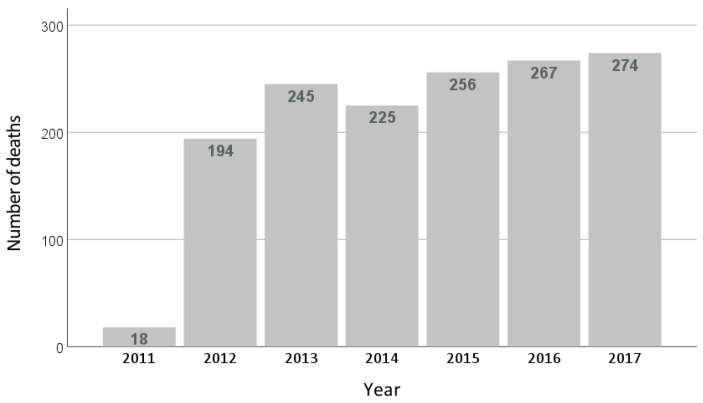
Distribution of mortality by year.

The CI of total mortality was 5.4% (n = 1107) with a maximum follow-up of 6.4 years (mean follow-up of 6.2 years, with a total of 127,711 subject-years of follow-up). The incidence of total mortality in the group that visited the physiotherapist the previous year was 3.5% (n = 74) compared with 5.6% (n = 1033) of those who did not visit the physiotherapist (*p* < 0.001). The RRc was 0.62 (95% CI: 0.50–0.79), the RRR was 0.38 (95% CI: 0.21–0.50), and the EIN was 47.1 (95% CI: 33.5–79.0). Table S3 shows other factors that were associated with all-cause mortality. Figure 2 shows the distribution of mortality by year.

In the multivariate analysis, the RRa of total mortality in the group that visited the physiotherapist was 0.699 (95% CI: 0.528–0.927; *p* = 0.013), which means that visiting the physiotherapist was associated with lower all-cause mortality (quantified at 30.1%) compared with not visiting the physiotherapist. This estimation was adjusted for 28 variables (Table 2). Table S4 shows the estimation of the relative risk of death of the complete multivariate model.

Table 1 and Table S2 show the profile of study participants according to whether they visited the physiotherapist in the previous year. People belonging to the age group between 55–64 years went to the physiotherapist the most (11.9%; n = 318; *p* < 0.001), while those that went to the physiotherapist the least were 85 years or older (4.4%; n = 21; *p* < 0.001). People with obesity went to the physiotherapist less than people with normal weight (8.7%; n = 272; *p* < 0.001 vs. 11.4%; n = 1027; *p* < 0.001). Level of income was directly proportional to attendance at the physiotherapist, with higher attendance in the group with incomes over EUR 2251 and lower attendance in the group with incomes under EUR 800 (15.4% n = 491; *p* < 0.001 vs. 6.3% n = 172; *p* < 0.001). People who slept less than 7 hours a day went to the physiotherapist more than those who slept more than 9 hours a day (13.0%; n = 589; *p* < 0.001 vs. 7.2%; n = 88; *p* < 0.001). Regarding leisure physical activity, sedentary people went to the physiotherapist less (8.5%; n = 770; *p* < 0.001) than those who performed physical activity frequently (13.8%; n = 368; *p* < 0.001) and those who performed sports training (17.4%; n = 337; *p* < 0.001). People who rated their health as poor (17.0%; n = 209; *p* < 0.001) or very poor (15.8%; n = 47; *p* < 0.001) went to the physiotherapist more than those who rated it as good (9.8%; n = 1017; *p* < 0.001) or very good (6.1%; n = 270; *p* < 0.001). Not having a chronic disease was associated with not having visited a physical therapist (8.5%; n = 1005; *p* < 0.001). Likewise, not having presented any physical limitation during the previous two weeks was also associated with not having visited the physiotherapist (9.2%; n = 1658; *p* < 0.001). Regarding the use of health services, people without hospital admission in the previous year (9.6%; n = 1788; *p* < 0.001), those who did not visit the general practitioner in the previous month (9.6%; n = 1408; *p* < 0.001) or the specialist physician (8.7%; n = 1513; *p* < 0.001), and those who did not attend a day hospital in the previous year (9.7%; n = 1836; *p* < 0.001) visited the physical therapist less than those who used these health services.

## 4. Discussion

The present study shows an association between visiting the physiotherapist and all-cause mortality at six years (quantified at 30% and adjusted for 28 variables from all the blocks of the survey: sociodemographic, lifestyle, health and comorbidities, and use of health services). In addition, this study shows that the factors associated with not visiting the physiotherapist were being aged 75 years or older, having obesity, having a low income (less than EUR 800), sleeping more than 9 hours, being sedentary, having a very good self-perceived health state, not suffering from a chronic illness or not having presented any physical limitation in the two weeks before being surveyed, and not having been admitted to hospital or visited a day hospital during the previous year or having visited the general practitioner or a specialist during the previous month.

Consistent with other previous works, this study identified differences regarding the profile of the physiotherapy patients, such as certain comorbidities, the operation of the health system making the physiotherapy service available, educational level, and the presence of serious conditions [24,25]. According to Anderson [26], variability in terms of healthcare would be appropriate when it is due to the patient’s clinical health condition, but it is questionable to consider whether variation is appropriate when it is due to factors such as social structure, beliefs about health, or accessibility. For example, our study found that people with low incomes visited the physiotherapist less than people with high incomes. This might be due to the saturation of the public health system and the limitation of paying for a private service [6]. Removing any inappropriate variation is necessary to improve the quality of physiotherapy care [27], and, for this, factors associated with not receiving physiotherapeutic care first need to be identified, as we have explored in the present study, in order to take steps in this regard and develop new strategies to improve access to this service.

Currently, increased participation in sports and physical exercise is widely promoted as an approach to a physically active lifestyle that has a positive effect on healthy aging. This has caused a higher incidence of sports-related injuries [28] and a greater presence of these patients at the physiotherapist’s consulting room as reflected in the results of this study. However, according to our results, sedentary and obese people visited the physiotherapist less than people with a normal weight.

In our study, working-age individuals went to the physiotherapist more often than the non-working-age population. According to other research, the working-age population exhibits a high incidence of musculoskeletal injuries, being the main reason for them to receive medical attention, hospitalisations, emergency visits, physical rehabilitation, and physiotherapy [29].

Some of the main physiotherapy interventions developed in the prevention and promotion of health and that may be related to improvement in certain health states that are indirectly associated with a reduction in all-cause mortality include:

(A) Improvement in self-perceived health and quality of life:

In a systematic review conducted in 2008, greater well-being was associated with lower all-cause mortality in initially healthy population cohorts (adjusted hazard ratio 0.82, 95% confidence interval 0.76 to 0.89) [30]. Subsequently, several studies documented protective associations between various measures of subjective well-being and mortality [31,32]. In the elderly with good mobility, this association may be irrespective of age, sex, educational level, marital status, and drug use [33].

Quality of life among the elderly becomes poorer as they get older, and, consequently, so does their subjective well-being, partly due to health deterioration with the appearance of chronic diseases and, especially, the progressive loss of functionality [34]. In this regard, the promotion of physical activity among the elderly plays a key role in healthy aging, possibly representing an impact beyond functionality, affecting mental health and quality of life. The WHO warns that physical inactivity is a major risk factor that causes the most deaths, ranking fourth behind HBP, tobacco, and hyperglycaemia [35]. This means that physical inactivity is the cause of 6% of deaths recorded worldwide. According to Blair [36], physical inactivity is one of the most important public health problems in the twentieth century. Physiotherapy might have a differential impact on the maintenance and improvement of self-perceived health through the promotion of health and well-being, prevention of limitations and restrictions on activity, and social participation in people with movement disorders [36]. According to this study, people who rated their health as poor or very poor visited the physiotherapist more often; this fact could be a turning point.

(B) Improvement of musculoskeletal disorders that may lead to decreased mobility:

Chronic degenerative musculoskeletal pathologies inherent in age constitute one of the fundamental causes of loss of functional independence. Being able to re-establish an adequate condition to maintain optimal and adapted functionality has proven to be essential in the maintenance of functional capacity and is closely related to life expectancy and mortality [37]. In this regard, physiotherapists are healthcare professionals, often the first choice and easily accessible in the private sector, trained for the management and proper treatment of musculoskeletal injuries [38]. This study reported that those patients who suffered from a chronic degenerative disease such as osteoarthritis, chronic cervical pain, chronic low back pain, or osteoporosis resorted to physiotherapy more regularly.

(C) Urgent referral to other health professionals:

In many cases, physiotherapists will be in charge of the treatment of musculoskeletal disorders or other types of injuries, prescribing adapted therapeutic exercises minimising or reducing the sedentary lifestyle caused by such disorders. Sometimes they will ensure quick referral to other healthcare professionals and detect red flags that require urgent attention [38]. A physiotherapist’s ability to identify clinical signs of danger (i.e., detection of red flags) and understand when patients should be referred to a physician is vital to patient safety [39]. It has been reported that physiotherapists diagnose with the same accuracy as orthopaedic surgeons, as sports medicine doctors [40], in patients with musculoskeletal disorders [41], and as general practitioners in the UK [42]. In some situations, physiotherapists can contribute to patient safety by identifying the presence of a wide range of systemic diseases and various pathologies requiring medical management.

(D) Fall prevention:

Falls represent one of the main causes of injury, functional impairment, repeated visits to the physician, readmission to hospital, and mortality in people aged 65 and over [43,44]. In addition, decreased mobility during hospitalisation is associated with loss of muscle mass, increased potential risk of falls, functional decline, and increased mortality, especially in the elderly [45,46]. Promoting mobility soon after an intervention potentially decreases the risk of falls [47]. Falls that do not result in physical injury can also have serious implications [48]. Exercise and physiotherapy interventions are shown to be very effective in preventing falls [44,49,50]. Despite this, people over 65 years visited the physiotherapist less than other age groups in this study. Thus, it is important to facilitate access to the physiotherapist for this age group.

Visiting the physiotherapist does not directly reduce mortality, but, in certain situations, it may modify intermediate health states that indirectly reduce total mortality, acting as a positive mediator between certain health/disease states and total mortality. Knowing the determinant factors related to physiotherapist access could help to develop intervention strategies to improve access to this type of health care and analyse whether these strategies are effective and can represent greater protection against mortality in physiotherapy patients through properly designed prospective studies.

### Limitations

The present study has some limitations that should be considered. The data on the exposure and explanatory study variables are from the respondents’ self-reported information, a fact that could generate a recall bias. However, previous studies that compared patients’ answers in questionnaires against the data from medical records showed a high degree of sensitivity and agreement [51]. Similarly, self-reported chronic diseases have been considered a source of useful information for prevalence studies and have been validated [52]. Likewise, the data were obtained from information collected in the ENSE16, which has national and regional representation and constitutes one of the largest data collection programs of the Spanish Ministry of Health, whose results have been validated and are considered an essential element of territorial cohesion for population monitoring. The official data from the INE have been analysed, being a comprehensive population sample for all the years under study [17]. However, the ENSE11 only provides information on attendance at physiotherapy treatment, but not on frequency, subsequent visits, or reasons for visiting the physiotherapist. Information about external factors, such as government education campaigns or changes in health spending, that might potentially have had an impact on mortality, were not included either in the study analysis.

## 5. Conclusions

Visiting the physiotherapist was associated with lower mortality from all causes in the population living in Spain. Factors associated with not visiting the physiotherapist were being 75 years of age or older; being obese; having a low income (less more than EUR 800); sleeping more than 9 h; being sedentary; having a very good self-perceived health state; not suffering from a chronic illness or not having presented any physical limitation in the two weeks prior to being surveyed; not having been admitted to hospital or visited a day hospital during the previous year; and not having visited the general practitioner or a specialist during the previous month.

## Figures and Tables

**Table 1:** The proportion of visits to the physiotherapist in the previous year according to the characteristics of the study sample.

		Total		No Physiotherapy		Physiotherapy		
				Visit (Previous Year)		Visit (Previous Year)		
		n	%	n	%	n	%	*p*-Value
Sex	Man	9942	48.7	8946	90	996	10	0.196
	Woman	10,455	51.3	9350	89.4	1105	10.6	
Age group	<35 years	6020	29.5	5429	90.2	591	9.8	<0.001
	35–54 years	7671	37.6	6781	88.4	890	11.6	
	55–64 years	2685	13.2	2367	88.1	318	11.9	
	65–74 years	2060	10.1	1892	91.8	168	8.2	
	75–84 years	1475	7.2	1364	92.5	111	7.5	
	≥85 years	485	2.4	464	95.6	21	4.4	
BMI	Normal	9019	44.2	7992	88.6	1027	11.4	<0.001
	Overweight	6803	33.4	6090	89.5	713	10.5	
	Obesity	3144	15.4	2871	91,3	272	8.7	
	NR/DK	1431	7	1343	93.8	88	6.2	
Monthly household income (net)	NR/DK	5441	26.7	4924	90.5	517	9.5	<0.001
	>EUR 2251	3195	15.7	2704	84.6	491	15.4	
	EUR 1551–2250	3206	15.7	2813	87.7	393	12.3	
	EUR 1051–1550	3765	18.5	3412	90.6	354	9.4	
	EUR 801–1050	2077	10.2	1904	91.7	173	8.3	
	<EUR 800	2713	13.3	2541	93.7	172	6.3	
Hours of sleep	>9 h/day	1226	6	1139	92.8	88	7.2	<0.001
	7–9 h/day	14,643	71.8	13,219	90.3	1424	9.7	
	<7 h/day	4528	22.2	3939	87	589	13	
Physical leisure activity	Sedentary	9051	44.4	8282	91.5	770	8.5	<0.001
	Occasional physical activity	6735	33	6110	90.7	625	9.3	
	Frequent physical activity	2668	13.1	2300	86.2	368	13.8	
	Sports training	1943	9.5	1605	82.6	337	17.4	
Self-perceived health	Very good	4396	21.6	4126	93.9	270	6.1	<0.001
	Good	10,413	51	9396	90.2	1017	9.8	
	Fair	4065	19.9	3508	86.3	557	13.7	
	Bad	1224	6	1016	83	209	17	
	Very bad	299	1.5	251	84.2	47	15.8	
Any chronic disease	No	11,823	58	10,818	91.5	1005	8.5	<0.001
	Yes	8574	42	7479	87.2	1095	12.8	
Activity limitation (previous 6 months)	Severely limited	672	3.3	544	80.9	129	19.1	<0.001
	Non-severe limited	3238	15.9	2621	81	617	19	
	Not limited	16,487	80.8	15,132	91.8	1355	8.2	
Hospital admission (previous year)	No	18,702	91.7	16,914	90.4	1788	9.6	<0.001
	Yes	1695	8.3	1383	81.6	312	18.4	
General practitioner visit (previous month)	No	14,678	72	13,270	90.4	1408	9.6	<0.001
	Yes	5719	28	5027	87.9	692	12.1	
Specialist visit (previous month)	No	17,330	85	15,817	91.3	1513	8.7	<0.001
	Yes	3067	15	2479	80.8	587	19.2	
Day hospital (previous year)	No	18,858	92.5	17,021	90.3	1836	9.7	<0.001
	Yes	1539	7.5	1275	82.9	264	17.1	

BMI: Body Mass Index.

**Table 2:** Adjusted relative risk of death at 6 years estimated by Poisson models with robust variance for visits to the physiotherapist in the previous year.

Physiotherapy Visit (Previous Year)	RRa *	95% CI	*p*-Value
No	1		
Yes	0.699	0.528–0.927	0.013

n train = 14,191; n° deaths train = 1040; LRT = 2102.2 (*p* < 0.001); n test = 6206; n° deaths test = 439; ROC area test = 0.921; 95% CI = (0.908–0.933). * Adjusted for sex, age, BMI, marital status, tobacco use, main daily activity, leisure physical activity, consumption of legumes, dental hygiene, net monthly household income, having a chronic disease, having had an acute myocardial infarction, having allergies, COPD, diabetes, high cholesterol, depression, malignant tumours, osteoporosis, activity limitations in the previous two weeks, hospital admission in the previous year, visit to a primary care doctor in the previous month, visit to a specialist doctor in the previous month, visit to a day hospital in the previous year, and having had a CT scan, ultrasound or MRI in the previous year.

## Data Availability

The data presented in this study are available on request from the corresponding author. Data can be downloaded for free [16,17].

## Supplementary Materials

The following supporting information can be downloaded at: https://www.mdpi.com/article/10.3390/medicina59122187/s1. Table S1. Details of the explanatory variables; Table S2. Descriptives of the sample and proportion of visits to the physiotherapist in the previous year according to explicative variables; Table S3. Cumulative incidence of total mortality at 6 years according to explanatory variables; Table S4. Relative risks estimated by Poisson models with robust variance.

## References

[1] World.physio. https://world.physio/sites/default/files/2021-05/PS-2019-Disability-Spanish.pdf.

[2] American Physical Therapy Association (2001). Guide to Physical Therapist Practice. Second Edition. American Physical Therapy Association. Phys. Ther..

[3] Pan American Health Association Carta de Ottawa para la Promoción de la Salud. https://www.paho.org/hq/dmdocuments/2013/Carta-de-ottawa-para-la-apromocion-de-la-salud-1986-SP.pdf.

[4] Gallego Izquierdo T. (2007). Bases Teóricas y Fundamentos de la Fisioterapia.

[5] Practising Physiotherapists, per 100,000—Eurostat Statistics Explaned. https://ec.europa.eu/eurostat/statistics-explained/index.php?title=File:Practising_physiotherapists,_2020_(per_100_0000_inhabitants)_Health2022.png.

[6] Fernández-Lago H., Climent-Sanz C., Bravo C., Bosch-Barceló P., Masbernat-Almenara M., Sanjuan-Sánchez D., Briones-Vozmediano E. (2023). Physiotherapists’ experiences on assisting physiotherapy users during the COVID-19 pandemic with lockdown measures in Spain. Physiother. Res. Int..

[7] Martínez-López J.Á., Martínez-Gayo G. (2019). Consequences of the Increase of the Pharmaceutical Copayment in Spain: A New Material Deprivation. Convergencia.

[8] Vos T., Lim S.S., Abbafati C., Abbas K.M., Abbasi M., Abbasifard M., Abbasi-Kangevari M., Abbastabar H., Abd-Allah F., Abdelalim A. (2020). Global burden of 369 Diseases and Injuries in 204 Countries and Territories, 1990–2019: A Systematic Analysis for the Global Burden of Disease Study 2019. Lancet.

[9] Hadjistavropoulos T., Herr K., Turk D.C., Fine P.G., Dworkin R.H., Helme R., Jackson K., Parmelee P.A., Rudy T.E., Beattie B.L. (2007). An interdisciplinary expert consensus statement on assessment of pain in older persons. Clin. J. Pain..

[10] Jadczak A.D., Makwana N., Luscombe-Marsh N., Visvanathan R., Schultz T.J. (2018). Effectiveness of exercise interventions on physical function in community-dwelling frail older people: An umbrella review of systematic reviews. JBI Database Syst. Rev. Implement. Rep..

[11] Fundadeps.org. https://fundadeps.org/wp-content/uploads/eps_media/recursos/documentos/267/libro_ejercicio_cardiosaludable_CSD.pdf.

[12] World Health Organization Musculoskeletal Health. https://www.who.int/es/news-room/fact-sheets/detail/musculoskeletal-conditions.

[13] Barger S.D., Broom T.W., Esposito M.V., Lane T.S. (2020). Is subjective well-being independently associated with mortality? A 14-year prospective cohort study in a representative sample of 25 139 US men and women. BMJ Open.

[14] Gentil P., de Lira C.A.B., Vieira C.A., Ramirez-Campillo R., Haghighi A.H., Clemente F.M., Souza D. (2022). Resistance Training before, during, and after COVID-19 Infection: What Have We Learned So Far?. Int. J. Environ. Res. Public Health.

[15] Momma H., Kawakami R., Honda T., Sawada S.S. (2022). Muscle-strengthening activities are associated with lower risk and mortality in major non-communicable diseases: A systematic review and meta-analysis of cohort studies. Br. J. Sports Med..

[16] National Institute of Statistics (2011). National Health Survey. https://www.ine.es/metodologia/t15/t153041912.pdf.

[17] National Institute of Statistics (2022). Deaths by Death Cause. https://www.ine.es/dyngs/INEbase/es/operacion.htm?c=Estadistica_C&cid=1254736176780&menu=ultiDatos&idp=1254735573175.

[18] Álvarez-Dardet C., Alonso J., Domingo A., Regidor (1995). La Medición de la Clase Social en Ciencias de la Salud.

[19] Heller R.F., Dobson A.J., Attia J., Page J. (2002). Impact numbers: Measures of risk factor impact on the whole population from case-control and cohort studies. J. Epidemiol. Community Health.

[20] Zou G. (2004). A modified poisson regression approach to prospective studies with binary data. Am. J. Epidemiol..

[21] Gómez-Beneyto M., Nolasco A., Moncho J., Pereyra-Zamora P., Tamayo-Fonseca N., Munarriz M., Salazar J., Tabarés-Seisdedos R., Girón M. (2013). Psychometric behaviour of the strengths and difficulties questionnaire (SDQ) in the Spanish national health survey 2006. BMC Psychiatry.

[22] IBM Corp (2021). IBM SPSS Statistics for Windows, Version 28.0, Released 2021.

[23] R Core Team (2020). R: A Language and Environment for Statistical Computing.

[24] Rabah N.M., Knusel K.D., Khan H.A., Marcus R.E. (2020). Are There Nationwide Socioeconomic and Demographic Disparities in the Use of Outpatient Orthopaedic Services?. Clin. Orthop. Relat. Res..

[25] Davis M.A., Onega T., Weeks W.B., Lurie J.D. (2012). Where the United States spends its spine dollars: Expenditures on different ambulatory services for the management of back and neck conditions. Spine.

[26] Anderson J.G. (1973). Health services utilization: Framework and review. Health Serv. Res..

[27] Hannan T.J. (1999). Variation in health care--the roles of the electronic medical record. Int. J. Med. Inf..

[28] Minoves Font M. (2020). Clinical applications of nuclear medicine in the diagnosis and assessment of musculoskeletal sports injuries. Rev. Esp. Med. Nucl. Imagen Mol. (Engl. Ed.).

[29] Ruesga S.M., Vasco D.C., Gómez V., Monsueto S.E., Nestares C.R., Bichara J.S. Work accident mutuals and the management of temporary disability [Internet]. Seg-social.es. https://www.seg-social.es/wps/wcm/connect/wss/1e437183-27ec-439b-afa1-4faa23c90f7c/F45_1_07.pdf?MOD=AJPERES.

[30] Chida Y., Steptoe A. (2008). Positive psychological well-being and mortality: A quantitative review of prospective observational studies. Psychosom. Med..

[31] Gana K., Broc G., Saada Y., Amieva H., Quintard B. (2016). Subjective wellbeing and longevity: Findings from a 22-year cohort study. J. Psychosom. Res..

[32] Lawrence E.M., Rogers R.G., Wadsworth T. (2015). Happiness and longevity in the United States. Soc. Sci. Med..

[33] Trudel-Fitzgerald C., Kubzansky L.D., VanderWeele T.J. (2021). A Review of Psychological Well-Being and Mortality Risk: Are All Dimensions of Psychological Well-Being Equal?. Interdisciplinary Perspectives from the Social Sciences and the Humanities.

[34] Kojima G., Iliffe S., Jivraj S., Walters K. (2016). Association between frailty and quality of life among community-dwelling older people: A systematic review and meta-analysis. J. Epidemiol. Community Health.

[35] World Health Organization Global Health Risks: Mortality and Burden of Disease Attributable to Selected Major Risks.

[36] Blair S.N. (2009). Physical inactivity: The biggest public health problem of the 21st century. Br. J. Sports Med..

[37] World Physiotherapy Policy Statement: Patient/Client Direct Access and Self-Referral to Physical Therapy [Internet]. http://www.wcpt.org/policy/ps-direct-access.

[38] Downie F., McRitchie C., Monteith W., Turner H. (2019). Physiotherapist as an alternative to a GP for musculoskeletal conditions: A 2-year service evaluation of UK primary care data. Br. J. Gen. Pr..

[39] Welch E. (2011). Red flags in medical practice. Clin. Med..

[40] Décary S., Fallaha M., Pelletier B., Frémont P., Martel-Pelletier J., Pelletier J.P., Feldman D.E., Sylvestre M.P., Vendittoli P.A., Desmeules F. (2017). Diagnostic validity and triage concordance of a physiotherapist compared to physicians’ diagnoses for common knee disorders. BMC Musculoskelet. Disord..

[41] Matifat E., Perreault K., Roy J.S., Aiken A., Gagnon E., Mequignon M., Lowry V., Décary S., Hamelin B., Ambrosio M. (2019). Concordance between physiotherapists and physicians for care of patients with musculoskeletal disorders presenting to the emergency department. BMC Emerg. Med..

[42] Vedanayagam M., Buzak M., Reid D., Saywell N. (2021). Advanced practice physiotherapists are effective in the management of musculoskeletal disorders: A systematic review of systematic reviews. Physiotherapy.

[43] Miró Ò., Nayla Brizzi B., Aguiló S., Alemany X., Jacob J., Llorens P., Herrero Puente P., González Ramón B., Castro Jiménez V., Torres Machado V. (2018). Profile of elderly patients seen in the emergency room due to falls (FALL-ER Registry): Magnitude of the problem and possibilities for improvement in hospital emergency services. Emergencies.

[44] Cáceres Santana E., Bermúdez Moreno C., Ramírez Suarez J., Bahamonde Román C., Murie-Fernández M. (2022). Incidence of falls in long-stay hospitals: Risk factors and strategies for prevention. Neurologia.

[45] Kanejima Y., Shimogai T., Kitamura M., Ishihara K., Izawa K.P. (2020). Effect of Early Mobilization on Physical Function in Patients after Cardiac Surgery: A Systematic Review and Meta-Analysis. Int. J. Environ. Res. Public Health.

[46] Coker R.H., Hays N.P., Williams R.H., Wolfe R.R., Evans W.J. (2015). Bed rest promotes reductions in walking speed, functional parameters, and aerobic fitness in older, healthy adults. J. Gerontol. A Biol. Sci. Med. Sci..

[47] Growdon M.E., Shorr R.I., Inouye S.K. (2017). The Tension Between Promoting Mobility and Preventing Falls in the Hospital. JAMA Intern. Med..

[48] Tinetti M.E., Williams C.S. (1998). The effect of falls and fall injuries on functioning in community-dwelling older persons. J. Gerontol. A Biol. Sci. Med. Sci..

[49] Montero-Odasso M.M., Kamkar N., Pieruccini-Faria F., Osman A., Sarquis-Adamson Y., Close J., Hogan D.B., Hunter S.W., Kenny R.A., Lipsitz L.A. (2021). Evaluation of Clinical Practice Guidelines on Fall Prevention and Management for Older Adults: A Systematic Review. JAMA Netw. Open.

[50] Karlsson L., Doe K., Gerry M., Moore B., Wingood M., Renfro M., Gell N. (2020). Outcomes of a Physical Therapist-Led, Statewide, Community-Based Fall Risk Screening. J. Geriatr. Phys. Ther..

[51] Mac Donald R., Baken L., Nelson A., Nichol K.L. (1999). Validation of self-report of influenza and pneumococcal vaccination status in elderly outpatients. Am. J. Prev. Med..

[52] Martin L.M., Leff M., Calonge N., Garrett C., Nelson D.E. (2000). Validation of self-reported chronic conditions and health services in a managed care population. Am. J. Prev. Med..

