# Supplementary material for: Association of Visiting the Physiotherapist with Mortality in the Spanish General Population: A Population-Based Cohort Study
Source: Medicina (Kaunas). 2023 Dec 16;59(12):2187. doi: 10.3390/medicina59122187 (PMC10744916; doi:10.3390/medicina59122187)
Supplement: Supplementary file 1 [file medicina-59-02187-s001.zip › medicina-2746069-supplementary.pdf]

Supplemental Material: Association of Visiting the Physiotherapist with Mortality in the Spanish General Population: A Population-Based Cohort Study

Table S1. Details of the explanatory variables

VARIABLE	Description	Code Question Survey	Response options	CATEGORIES of response
Autonomous community residence	This variable is not coded in the National Health Survey 20112012. The enumerator has accessed this variable from other registers.			Andalucía<br>Aragón<br>Asturias<br>Baleares<br>Canarias<br>Cantabria<br>Castilla y León<br>Castilla-La Mancha<br>Cataluña<br>Comunitat Valenciana<br>Extremadura<br>Galicia<br>Madrid<br>Murcia<br>Navarra<br>País Vasco<br>Rioja, La<br>Ceuta<br>Melilla
Sex		Proxy_3b	1.- Man	Man

			2.-Woman	Woma
Age group	Age has been categorised into the intervals shown in the last column, with a total of six intervals.	Proxy_4	Free field	< 35 years 35-54 years 55-64 years 65-74 years 75-84 years >= 85 years
Occupational social	The definition proposed for each response category is as follows:<br><br>I. Managers in the Public Administration and in companies with 10 or more employees. Professions associated with second and third cycle university degrees.<br><br>II. Managers of companies with fewer than 10 employees. Professions associated with a first-cycle university degree. Technicians and support professionals. Artists and athletes.<br><br>IIIa. Administrative type employees and administrative and financial management support professionals. Personal and security services workers.			Social class I class contain a code in the variable constructed by NSI

	IIIb. Self-employed workers<br><br>IIIc. Supervisors of manual workers<br><br>IVa. Skilled manual workers<br><br>IVb Semi-skilled manual workers IVb.			
	V. Unskilled workers.			Social class II<br>Social class III<br>Social class IV<br>Social class V<br>Social class VI<br>No record
BMI	For the calculation of this variable, the answers to questions 102 and 103 contained in the Health Determinants Module (R-Physical Characteristics) of the National Health Survey 2011-2012 will be considered.	102<br><br>103	Free field	Normal<br><br>Overweight<br>Obesity<br>NR/DK
Country of birth	The response category is of the nominal type for this variable.	2	Spanish<br>Foreign<br>Doesn't know<br>No response	Spain<br><br>Foreign

Marital status	The response category is of the nominal type for this variable.	4	Single<br>Married<br>Widowed<br>Legally separated<br>Divorced<br>Doesn't know<br>No response	Single<br>Married<br>Widowed<br>Separated<br>Divorced
Education Level	.This variable is not coded in the National Health Survey 2011-2012. .<br>The enumerator has accessed this variable from other registers. This variable has been categorised into the survey modes noted in the last column.			University<br>VT<br>Baccalaurate<br>Secondary school<br>Primary or less
Tobacco consumption	The response category is of the nominal type for this variable. The response categories are mutually exclusive. See in the last column.	105	Yes, smokes daily<br>Smokes but not daily<br>Doesn't smoke now, but has smoked before<br>Doesn't smoke nor has ever smoked on a fair basis<br>Doesn't know<br>No response	Never<br>Ex-smoker<br>Smoker
Exposure to tobacco		117	Never or hardly ever<br>Less than one hour a day<br>Between 1 and 5 hours a day<br>More than 5 hours a day	Never
smoke	The response category is of the frequency interval type for this variable. See in the last column.			< 1h/day<br>> 1h/day

Alcohol risk		123	Everyday<br>From 3 to 6 times a week<br>From once to twice a week<br>From twice to 3 times a month<br>Once a month or less	Doesn't drink
	The response category is of the quantity range type for this variable. See in the last column.			Low risk < 20/40 gr<br>Risk 20/40 – 40/60 gr High risk > 40/60 gr
Tobacco consumption	The response category is of the nominal type for this variable. The response categories are mutually exclusive. See in the last column.	105	Yes, smokes daily<br>Smokes but not daily<br>Doesn't smoke now, but has smoked before<br>Doesn't smoke nor has ever smoked on a fair basis<br>Doesn't know<br>No response	Never<br>Ex-smoker<br>Smoker
Exposure to tobacco		117	Never or hardly ever<br>Less than one hour a day<br>Between 1 and 5 hours a day<br>More than 5 hours a day	Never
smoke	The response category is of the frequency interval type for this variable. See in the last column.			< 1h/day<br>> 1h/day

Alcohol risk		123	Everyday<br>From 3 to 6 times a week<br>From once to twice a week<br>From twice to 3 times a month<br>Once a month or less	Doesn’t drink
	The response category is of the quantity range type for this variable. See in the last column.			Low risk < 0/40 gr<br>Risk 20/40 – 40/60 gr High risk > 40/60 gr
Daily main activity	The response category is of the nominal type for this variable. See in the last column.	128	Sitting most of the day.<br>Standing most of the day without much movement or effort.<br>Walking, carrying some weight, making frequent movements.<br>Performing tasks that require great physical effort.<br>Doesn't know<br>No response	Sitting most of the time<br>Standing most of the time<br>Walking with weight<br>Tasks with effort

Leisure physical activity	The response category is of the nominal type for this variable. See in the last column.	129	I do exercise<br>I do some physical or sports activity occasionally<br>I do physical activity several times a month<br>I do sports or physical training several times a week<br>Doesn't know<br>No response	Sedentary<br>Occasional physical activity<br>Frequent physical activity<br>Sports training
Breakfast	The response category is of the nominal type for this variable. See in the last column.	131	At home, before going out<br>Away from home<br>Don't usually have<br>breakfast	At home<br>Away from home Don't usually have breakfast
Fruit consumption	The response category for this variable is frequency intervals. See the frequency intervals in the last column.	133	Multiple choice table	Daily<br>> 3 times/week once-twice/week < once/week never or hardly ever

Vegetables consumption	The response category for this variable is frequency intervals. See the frequency intervals in the last column.	133	Multiple choice table	daily<br>> 3 times/week once-twice/week < once/week never or hardly ever
Legume consumption	The response category for this variable is frequency intervals. See the frequency intervals in the last column.	133	Multiple choice table	daily<br>> 3 times/week once-twice/week < once/week never or hardly ever
Dairy consumption	The response category for this variable is frequency intervals. See the frequency intervals in the last column.	133	Multiple choice table	daily<br>> 3 times /week once-twice/week < once/week n ever o ardly ever
Fast food	The response category for this variable is frequency intervals. See the frequency intervals in the last column.	133	Multiple choice table	daily<br>> 3 times/week once-twice/week < once/week never or hardly ever

Dental hygiene	The response category for this variable is frequency intervals. See the frequency intervals in the last column.	137	Occasionally, not every day Never<br>Once a day<br>Twice a day<br>Three or more times a day<br>Doesn't know<br>No response	3 or more times<br>/ day twice/day<br>once/day<br>never or occasionally
Self-perceived health	The response category is of the nominal type for this variable. See in the last column.	26.6	Response scale	Very good<br>Good<br>Fair<br>Bad<br>Very bad
Any chronic disease	The response category is of the nominal type for this variable. See in the last column.	21 a	Yes<br>No<br>Doesn't know<br>No response	No<br>Yes

AMI	The response category is of the nominal type for this variable. See in the last column.	21 a	Yes<br>No<br>Doesn't know<br>No response	No<br><br>Yes
Other heart diseases	The response category is of the nominal type for this variable. See in the last column.	21 a	Yes<br>No<br>Doesn't know<br>No response	No<br><br>Yes
Varicose veins	The response category is of the nominal type for this variable. See in the last column.	21 a	Yes<br>No<br>Doesn't know<br>No response	No<br><br>Yes
Osteoarthritis	The response category is of the nominal type for this variable. See in the last column.	21 a	Yes<br>No<br>Doesn't know<br>No response	No<br><br>Yes

Chronic neck pain	The response category is of the nominal type for this variable. See in the last column	21 a	Yes<br>No<br>Doesn't know<br>No response	No<br>Yes
Chronic low back pain	The response category is of the nominal type for this variable. See in the last column.	21 a	Yes<br>No<br>Doesn't know<br>No response	No<br>Yes
Allergy	The response category is of the nominal type for this variable. See in the last column.	21 a	Yes<br>No<br>Doesn't know<br>No response	No<br>Yes
Asthma	The response category is of the nominal type for this variable. See in the last column.	21 a	Yes<br>No<br>Doesn't know<br>No response	No<br>Yes
COPD	The response category is of the nominal type for this variable. See in the last column.	21 a	Yes<br>No<br>Doesn't know<br>No response	No<br>Yes
Diabetes Mellitus	The response category is of the nominal type for this variable. See in the last column.	21 a	Yes<br>No<br>Doesn't know<br>No response	No<br>Yes
Stomach ulcer	The response category is of the nominal type for this variable. See in the last column.	21 a	Yes<br>No<br>Doesn't know<br>No response	No<br>Yes

Urinary incontinence	The response category is of the nominal type for this variable. See in the last column.	21 a	Yes<br>No<br>Doesn't know<br>No response	No<br>Yes
High cholesterol	The response category is of the nominal type for this variable. See in the last column.	21 a	Yes<br>No<br>Doesn't know<br>No response	No<br>Yes
Cataracts	The response category is of the nominal type for this variable. See in the last column.	21 a	Yes<br>No<br>Doesn't know<br>No response	No<br>Yes
Skin problems	The response category is of the nominal type for this variable. See in the last column.	21 a	Yes<br>No<br>Doesn't know<br>No response	No<br>Yes
Chronic constipation	The response category is of the nominal type for this variable. See in the last column.	21 a	Yes<br>No<br>Doesn't know<br>No response	No<br>Yes
Cirrhosis	The response category is of the nominal type for this variable. See in the last column.	21 a	Yes<br>No<br>Doesn't know<br>No response	No<br>Yes
Depression	The response category is of the nominal type for this variable. See in the last column.	21 a	Yes<br>No<br>Doesn't know<br>No response	No<br>Yes

Anxiety	The response category is of the nominal type for this variable. See in the last column.	21 a	Yes<br>No<br>Doesn't know<br>No response	No<br>Yes
Other mental problems	The response category is of the nominal type for this variable. See in the last column.	21 a	Yes<br>No<br>Doesn't know<br>No response	No<br>Yes
Ictus	The response category is of the nominal type for this variable. See in the last column.	21 a	Yes<br>No<br>Doesn't know<br>No response	No<br>Yes
Migraine	The response category is of the nominal type for this variable. See in the last column.	21 a		No<br>Yes
Haemorrhoids	The response category is of the nominal type for this variable. See in the last column.	21 a	Yes<br>No<br>Doesn't know<br>No response	No<br>Yes
Malignant tumours	The response category is of the nominal type for this variable. See in the last column.	21 a	Yes<br>No<br>Doesn't know<br>No response	No<br>Yes
Osteoporosis	The response category is of the nominal type for this variable. See in the last column.	21 a	Yes<br>No<br>Doesn't know<br>No response	No<br>Yes

Thyroid problems	The response category is of the nominal type for this variable. See in the last column.	21 a	Yes<br>No<br>Doesn't know<br>No response	No<br>Yes
Injuries or accidents	The response category is of the nominal type for this variable. See in the last column.	21 a	Yes<br>No<br>Doesn't know<br>No response	No<br>Yes
Accidents<br>(last year)	The response category is of the nominal type for this variable. See in the last column.	21 a	Yes<br>No<br>Doesn't know<br>No response	No<br>Yes
GHQ12 mental health	The response category is of the nominal type for this variable. See in the last column.	P.40	Multiple choice table	Absence of psychopathology<br>Suspicion<br>Presence of psychopathology<br>NR/DK
Activity limitation (last 2 weeks)	The response category is of the nominal type for this variable. See in the last column.	P.27	Free field<br>Yes<br>No	No<br>Yes
Bed rest (last 2 weeks)	The response category is of the nominal type for this variable. See in the last column.	P.28	Free field<br>Yes<br>No<br>Doesn't know<br>No response	No<br>Yes
Use of glasses or contact lenses	The response category is of the nominal type for this variable. See in the last column.	32	Yes<br>No, never<br>I’m blind and I can’t see at all<br>Doesn't know<br>No response	No<br>Yes

Table S2. Descriptives of the sample and proportion of visits to the physiotherapist in the last year according to explicative variables.

		Total		No Physiotherapy visit (last year)		Physiotherapy Visit (last year)		
		n	%	n	%	n	%	p-value
Autonomous community residence	Andalucía	3659	17.9 %	3418	93.4 %	241	6.6 %	**<0.001**
	Aragón	588	2.9 %	518	88.0 %	71	12.0 %	
	Asturias	485	2.4 %	415	85.5 %	70	14.5 %	
	Baleares	477	2.3 %	439	92.0 %	38	8.0 %	
	Canarias	931	4.6 %	827	88.9 %	103	11.1 %	
	Cantabria	263	1.3 %	252	95.9 %	11	4.1 %	
	Castilla y León	1143	5.6 %	1007	88.1 %	136	11.9 %	
	Castilla - La Mancha	918	4.5 %	846	92.1 %	72	7.9 %	
	Cataluña	3068	15.0 %	2722	88.7 %	347	11.3 %	
	Comunitat Valenciana	2233	10.9 %	1998	89.5 %	235	10.5 %	
	Extremadura	481	2.4 %	448	93.2 %	33	6.8 %	
	Galicia	1254	6.1 %	1117	89.1 %	137	10.9 %	
	Madrid	2804	13.7 %	2435	86.8 %	369	13.2 %	
	Murcia	649	3.2 %	560	86.3 %	89	13.7 %	
	Navarra	278	1.4 %	253	90.9 %	25	9.1 %	
	País Vasco	961	4.7 %	855	88.9 %	107	11.1 %	
	Rioja, La	141	0.7 %	128	90.9 %	13	9.1 %	

	Ceuta	30	0.1 %	28	91.9 %	2	8.1 %	
	Melilla	31	0.2 %	31	99.4 %	0	0.6 %	
Municipality size	>500,000 inhab.	3373	16.5 %	2980	88.4 %	393	11.6 %	**<0.001**
	Province capital	3333	16.3 %	2985	89.6 %	348	10.4 %	
	>100,000 inhab.	1992	9.8 %	1766	88.7 %	226	11.3 %	
	50,000 to 100,000 inhab.	2055	10.1 %	1869	90.9 %	186	9.1 %	
	20,000 to 50,000 inhab.	3166	15.5 %	2829	89.4 %	337	10.6 %	
	10,000 to 20,000 inhab.	2180	10.7 %	1928	88.5 %	252	11.5 %	
	<10,000 inhabitants	4298	21.1 %	3940	91.7 %	359	8.3 %	
Sex	Man	9942	48.7 %	8946	90.0 %	996	10.0 %	0.196
	Woman	10455	51.3 %	9350	89.4 %	1105	10.6 %	
Age group	< 35 years	6020	29.5 %	5429	90.2 %	591	9.8 %	**<0.001**
	35-54 years	7671	37.6 %	6781	88.4 %	890	11.6 %	
	55-64 years	2685	13.2 %	2367	88.1 %	318	11.9 %	
	65-74 years	2060	10.1 %	1892	91.8 %	168	8.2 %	
	75-84 years	1475	7.2 %	1364	92.5 %	111	7.5 %	
	>=85 years	485	2.4 %	464	95.6 %	21	4.4 %	
Occupational social class	Social class I	2185	10.7 %	1869	85.5 %	316	14.5 %	**<0.001**
	Social class II	1498	7.3 %	1270	84.8 %	228	15.2 %	
	Social class III	3646	17.9 %	3192	87.6 %	453	12.4 %	
	Social class IV	3046	14.9 %	2738	89.9 %	308	10.1 %	
	Social class V	6482	31.8 %	5924	91.4 %	558	8.6 %	
	Social class VI	3003	14.7 %	2811	93.6 %	192	6.4 %	

	No record	538	2.6 %	493	91.6 %	45	8.4 %	
BMI	Normal	9019	44.2 %	7992	88.6 %	1027	11.4 %	**<0.001**
	Overweight	6803	33.4 %	6090	89.5 %	713	10.5 %	
	Obesity	3144	15.4 %	2871	91,3 %	272	8.7 %	
	NR/DK	1431	7.0 %	1343	93.8 %	88	6.2 %	
Country of birth	Spain	17470	85.7 %	15513	88.8 %	1957	11.2 %	**<0.001**
	Foreign	2927	14.3 %	2784	95.1 %	143	4.9 %	
Marital status	Single	6573	32.2 %	5900	89.8 %	672	10.2 %	0.002
	Married	11398	55.9 %	10205	89.5 %	1193	10.5 %	
	Widowed	1460	7.2 %	1345	92.2 %	115	7.8 %	
	Separated	364	1.8 %	325	89.3 %	39	10.7 %	
	Divorced	604	3.0 %	522	86.5 %	82	13.5 %	
Education level	University	3280	16.1 %	2822	86.0 %	458	14.0 %	**<0.001**
	VT	3126	15.3 %	2742	87.7 %	384	12.3 %	
	Baccalaureate	2680	13.1 %	2396	89.4 %	284	10.6 %	
	Secondary school	6848	33.6 %	6179	90.2 %	669	9.8 %	
	Primary or less	4462	21.9 %	4156	93.1 %	306	6.9 %	
Monthly household income	NR/DK	5441	26.7 %	4924	90.5 %	517	9.5 %	**<0.001**
(net)	> € 2251	3195	15.7 %	2704	84.6 %	491	15.4 %	
	€ 1551-2250	3206	15.7 %	2813	87.7 %	393	12.3 %	
	€ 1051-1550	3765	18.5 %	3412	90.6 %	354	9.4 %	
	€ 801-1050	2077	10.2 %	1904	91.7 %	173	8.3 %	
	< € 800	2713	13.3 %	2541	93.7 %	172	6.3 %	
Tobacco consumption	Never	10908	53.5 %	9868	90.5 %	1040	9.5 %	**<0.001**

	Ex-smoker	3971	19.5 %	3512	88.4 %	460	11.6 %	
	Smoker	5518	27.1 %	4917	89.1 %	601	10.9 %	
Exposure to tobacco smoke	Never	16250	79.7 %	14594	89.8 %	1656	10.2 %	0.235
	<1h/day	1567	7.7 %	1386	88.5 %	181	11.5 %	
	>1h/day	2581	12.7 %	2318	89.8 %	263	10.2 %	
Alcohol risk	Non-drinker	10857	53.2 %	9840	90.6 %	1017	9.4 %	**<0.001**
	Low risk < 20/40 gr	9029	44.3 %	7984	88.4 %	1045	11.6 %	
	Risk 20/40-40/60 gr	439	2.2 %	404	92.0 %	35	8.0 %	
	High risk > 40/60 gr	72	0.4 %	69	95.3 %	3	4.7 %	
Hours of sleep	> 9h/day	1226	6.0 %	1139	92.8 %	88	7.2 %	**<0.001**
	7-9h/day	14643	71.8 %	13219	90.3 %	1424	9.7 %	
	<7h/day	4528	22.2 %	3939	87.0 %	589	13.0 %	
Daily main activity	Sitting most of the time	8161	40.0 %	7304	89.5 %	856	10.5 %	0.119
	Standing most of the time	9199	45.1 %	8250	89.7 %	948	10.3 %	
	Walking with weight	2481	12.2 %	2254	90.8 %	228	9.2 %	
	Strenuous tasks	556	2.7 %	489	87.8 %	68	12.2 %	
Leisure physical activity	Sedentary	9051	44.4 %	8282	91.5 %	770	8.5 %	**<0.001**
	Occasional physical activity	6735	33.0 %	6110	90.7 %	625	9.3 %	
	Frequent physical activity	2668	13.1 %	2300	86.2 %	368	13.8 %	
	Sports training	1943	9.5 %	1605	82.6 %	337	17.4 %	

Breakfast	At home	17288	84.8 %	15536	89.9 %	1752	10.1 %	0.036
	Away from home	2335	11.4 %	2060	88.2 %	275	11.8 %	
	Don't usually have breakfast	774	3.8 %	701	90.5 %	73	9.5 %	
Fruit consumption	Daily	12427	60.9 %	11039	88.8 %	1387	11.2 %	**<0.001**
	> 3 times/week	3957	19.4 %	3611	91.3 %	346	8.7 %	
	1-2 times/week	2274	11.1 %	2055	90.4 %	219	9.6 %	
	< once/week	787	3.9 %	724	92.0 %	63	8.0 %	
	Never or hardly ever	953	4.7 %	868	91.1 %	85	8.9 %	
Vegetables consumption	Daily	9566	46.9 %	8439	88.2 %	1128	11.8 %	**<0.001**
	> 3 times/week	6990	34.3 %	6333	90.6 %	657	9.4 %	
	1-2 times/week	2761	13.5 %	2553	92.5 %	208	7.5 %	
	< once/week	690	3.4 %	618	89.6 %	72	10.4 %	
	Never or hardly ever	390	1.9 %	355	91.0 %	35	9.0 %	
Legume consumption	Daily	312	1.5 %	293	93.8 %	19	6.2 %	**<0.001**
	> 3 times/week	4738	23.2 %	4325	91.3 %	413	8.7 %	
	1-2 times/week	12161	59.6 %	10871	89.4 %	1289	10.6 %	
	< once/week	2400	11.8 %	2105	87.7 %	294	12.3 %	
	Never or hardly ever	786	3.9 %	702	89.3 %	84	10.7 %	
Dairy consumption	Daily	17231	84.5 %	15410	89.4 %	1822	10.6 %	0.005
	> 3 times/week	1557	7.6 %	1439	92.4 %	118	7.6 %	
	1-2 times/week	702	3.4 %	638	90.8 %	65	9.2 %	

	< once/week	348	1.7 %	312	89.8 %	36	10.2 %	
	Never or hardly ever	559	2.7 %	498	89.1 %	61	10.9 %	
Sweet consumption	Daily	5924	29.0 %	5208	87.9 %	716	12.1 %	**<0.001**
	> 3 times/week	3105	15.2 %	2822	90.9 %	283	9.1 %	
	1-2 times/week	3708	18.2 %	3359	90.6 %	349	9.4 %	
	< once/week	3278	16.1 %	2935	89.5 %	343	10.5 %	
	Never or hardly ever	4382	21.5 %	3973	90.7 %	409	9.3 %	
Fast food	Daily	360	1.8 %	315	87.5 %	45	12.5 %	0.093
	> 3 times/week	919	4.5 %	835	90.9 %	84	9.1 %	
	1-2 times/week	4078	20.0 %	3683	90.3 %	395	9.7 %	
	< once/week	4861	23.8 %	4379	90.1 %	482	9.9 %	
	Never or hardly ever	10180	49.9 %	9085	89.2 %	1095	10.8 %	
Dental hygiene	3 or more times/day	7758	38.0 %	6871	88.6 %	887	11.4 %	**<0.001**
	twice/day	6738	33.0 %	6038	89.6 %	700	10.4 %	
	once/day	4034	19.8 %	3633	90.1 %	401	9.9 %	
	Never or occasionally	1866	9.1 %	1754	94.0 %	112	6.0 %	
Self-perceived health	Very good	4396	21.6 %	4126	93.9 %	270	6.1 %	**<0.001**
	Good	10413	51.0 %	9396	90.2 %	1017	9.8 %	
	Fair	4065	19.9 %	3508	86.3 %	557	13.7 %	
	Bad	1224	6.0 %	1016	83.0 %	209	17.0 %	
	Very bad	299	1.5 %	251	84.2 %	47	15.8 %	

Any chronic disease	No	11823	58.0 %	10818	91.5 %	1005	8.5 %	**<0.001**
	Yes	8574	42.0 %	7479	87.2 %	1095	12.8 %	
AH	No	16119	79.0 %	14425	89.5 %	1693	10.5 %	0.058
	Yes	4278	21.0 %	3871	90.5 %	407	9.5 %	
AMI	No	19997	98.0 %	17937	89.7 %	2060	10.3 %	0.960
	Yes	400	2.0 %	360	89.9 %	41	10.1 %	
Other heart disease	No	19226	94.3 %	17233	89.6 %	1993	10.4 %	0.182
	Yes	1171	5.7 %	1063	90.8 %	107	9.2 %	
Varicose veins	No	18087	88.7 %	16310	90.2 %	1777	9.8 %	**<0.001**
	Yes	2310	11.3 %	1987	86.0 %	323	14.0 %	
Osteoarthritis	No	16663	81.7 %	15096	90.6 %	1568	9.4 %	**<0.001**
	Yes	3734	18.3 %	3201	85.7 %	532	14.3 %	
Chronic neck pain	No	17053	83.6 %	15621	91.6 %	1432	8.4 %	**<0.001**
	Yes	3344	16.4 %	2675	80.0 %	669	20.0 %	
Chronic low back pain	No	16427	80.5 %	15020	91.4 %	1408	8.6 %	**<0.001**
	Yes	3970	19.5 %	3277	82.6 %	692	17.4 %	
Allergy	No	17921	87.9 %	16194	90.4 %	1727	9.6 %	**<0.001**
	Yes	2476	12.1 %	2103	84.9 %	373	15.1 %	
Asthma	No	19359	94.9 %	17381	89.8 %	1979	10.2 %	0.114
	Yes	1038	5.1 %	916	88.3 %	122	11.7 %	
COPD	No	19459	95.4 %	17463	89.7 %	1997	10.3 %	0.417
	Yes	938	4.6 %	834	89.0 %	104	11.0 %	

Diabetes mellitus	No	18932	92.8 %	16961	89.6 %	1971	10.4 %	0.051
	Yes	1465	7.2 %	1336	91.2 %	129	8.8 %	
Stomach ulcer	No	19545	95.8 %	17544	89.8 %	2001	10.2 %	0.194
	Yes	852	4.2 %	753	88.4 %	99	11.6 %	
Urinary incontinence	No	19710	96.6 %	17696	89.8 %	2014	10.2 %	0.051
	Yes	687	3.4 %	601	87.5 %	86	12.5 %	
High cholesterol	No	16617	81.5 %	14954	90.0 %	1663	10.0 %	0.005
	Yes	3780	18.5 %	3343	88.4 %	437	11.6 %	
Cataracts	No	18779	92.1 %	16824	89.6 %	1955	10.4 %	0.067
	Yes	1618	7.9 %	1472	91.0 %	145	9.0 %	
Skin problems	No	19432	95.3 %	17474	89.9 %	1958	10.1 %	**<0.001**
	Yes	965	4.7 %	823	85.3 %	142	14.7 %	
Chronic constipation	No	19572	96.0 %	17596	89.9 %	1975	10.1 %	**<0.001**
	Yes	825	4.0 %	701	84.9 %	125	15.1 %	
Cirrhosis	No	20195	99.0 %	18129	89.8 %	2066	10.2 %	**<0.001**
	Yes	202	1.0 %	168	82.9 %	35	17.1 %	
Depression	No	18918	92.7 %	17043	90.1 %	1874	9.9 %	**<0.001**
	Yes	1479	7.3 %	1254	84.7 %	226	15.3 %	
Anxiety	No	18836	92.3 %	16996	90.2 %	1839	9.8 %	**<0.001**
	Yes	1561	7.7 %	1300	83.3 %	261	16.7 %	
Other mental problems	No	20109	98.6 %	18036	89.7 %	2072	10.3	0.732
	Yes	288	1.4 %	261	90.4 %	28	9.6 %	

Ictus	No	20163	98.9 %	18097	89.8 %	2066	10.2 %	0.032
	Yes	234	1.1 %	200	85.5 %	34	14.5 %	
Migraine	No	18419	90.3 %	16634	90.3 %	1784	9.7 %	<0.001
	Yes	1978	9.7 %	1662	84.0 %	316	16.0 %	
Haemorrhoids	No	19200	94.1 %	17310	90.2 %	1891	9.8 %	<0.001
	Yes	1197	5.9 %	987	82.5 %	210	17.5 %	
Malignant tumours	No	19822	97.2 %	17794	89.8 %	2028	10.2 %	0.057
	Yes	575	2.8 %	503	87.4 %	73	12.6 %	
Osteoporosis	No	19570	95.9 %	17641	90.1%	1929	9.9 %	**<0.001**
	Yes	827	4.1 %	656	79.3 %	172	20.7 %	
Thyroid problems	No	19324	94.7 %	17385	90.0 %	1939	10.0 %	**<0.001**
	Yes	1073	5.3 %	911	85.0 %	161	15.0 %	
Permanent injuries due to accidents	No	19300	94.6 %	17454	90.4 %	1846	9.6 %	**<0.001**
	Yes	1097	5.4 %	843	76.9 %	254	23.1 %	
Accidents	No	18699	91.7 %	17035	91.1 %	1665	8.9 %	**<0.001**
(last year)	Yes	1698	8.3 %	1262	74.4 %	435	25.6 %	
GHQ12 mental health	Absence of psychopathology	17796	87.2 %	16048	90.2 %	1748	9.8 %	**<0.001**
	Suspicion	994	4.9 %	872	87.8 %	121	12.2 %	
	Presence of psychopathology	1367	6.7 %	1169	85.5 %	198	14.5 %	
	NR/DK	240	1.2 %	208	86.3 %	33	13.7 %	

Activity limitation	No	18077	88.6 %	16419	90.8%	1658	9.2 %	**<0.001**
(last 2 weeks)	Yes	2320	11.4 %	1878	80.9 %	442	19.1 %	
Bed rest	No	19297	94.6 %	17372	90.0 %	1925	10.0 %	**<0.001**
(last 2 weeks)	Yes	1100	5.4 %	925	84.0 %	176	16.0 %	
Activity limitation	Severely limited	672	3.3 %	544	80.9 %	129	19.1 %	**<0.001**
(last 6 months)	Non-severe limited	3238	15.9 %	2621	81.0 %	617	19.0 %	
	Not limited	16487	80.8 %	15132	91.8 %	1355	8.2 %	
Use of glasses or contact lenses	No	7953	39.0 %	7235	91.0 %	718	9.0 %	**<0.001**
	Yes	12444	61.0 %	11062	88.9 %	1382	11.1 %	
Use of glasses or contact lenses	No	19764	96.9 %	17738	89.8 %	2026	10.2 %	0.198
Use of hearing aid	Yes	633	3.1 %	559	88.2 %	75	11.8 %	
Hospital admission	No	18702	91.7 %	16914	90.4 %	1788	9.6 %	**<0.001**
(last year)	Yes	1695	8.3 %	1383	81.6 %	312	18.4 %	
General practitioner visit	No	14678	72.0 %	13270	90.4 %	1408	9.6 %	**<0.001**
(last month)	Yes	5719	28.0 %	5027	87.9 %	692	12.1 %	
Specialist visit	No	17330	85.0 %	15817	91.3 %	1513	8.7 %	**<0.001**
(last month)	Yes	3067	15.0 %	2479	80.8 %	587	19.2 %	
Day hospital	No	18858	92.5 %	17021	90.3 %	1836	9.7 %	**<0.001**
(last year)	Yes	1539	7.5 %	1275	82.9 %	264	17.1 %	

Emergency visits	No	14926	73.2 %	13735	92.0 %	1191	8.0 %	**<0.001**
(last year)	Yes	5471	26.8 %	4562	83.4 %	910	16.6 %	
Psychologist visit	No	19700	96.6 %	17769	90.2 %	1931	9.8 %	**<0.001**
(last year)	Yes	697	3.4 %	528	75.7 %	169	24.3 %	
X-ray	No	14831	72.7 %	13961	94.1 %	870	5.9 %	**<0.001**
(last year)	Yes	5566	27.3 %	4335	77.9 %	1230	22.1 %	
CT scan	No	18861	92.5 %	17126	90.8 %	1735	9.2 %	**<0.001**
(last year)	Yes	1536	7.5 %	1171	76.2 %	365	23.8 %	
Ultrasound	No	17472	85.7 %	15885	90.9 %	1586	9.1 %	**<0.001**
(last year)	Yes	2925	14.3 %	2411	82.4 %	514	17.6 %	
MRI	No	18884	92.6 %	17275	91.5 %	1608	8,5 %	**<0.001**
(last year)	Yes	1513	7.4 %	1021	67.5 %	492	32.5 %	
Influenza vaccine	No	16610	81.4 %	14875	89.6 %	1736	10.4 %	**<0.001**
(last campaign)	Yes	3787	18.6 %	3422	90.4 %	365	9.6 %	

BMI: Body Mass Index; VT: Vocational Training; AH: Arterial hypertension; AMI: Acute Myocardial Infarction; COPD: Chronic Obstructive Pulmonary Disease; GHQ: General Health Questionnaire; CT scan: Computed Axial Tomography; MRI: Magnetic Resonance Imaging.

Table S3. Cumulative incidence of total mortality at 6 years according to explanatory variables.

		Éxitus No		Éxitus Yes		p-value
		n	%	n	%	
Physiotherapist (last year)	No	17264	94.4 %	1033	5.6 %	**<0.001**
	Yes	2026	96.5 %	74	3.5 %	
Autonomous community	Andalaucía	3431		227<br>36<br>38<br>24<br>44<br>12<br>91<br>55<br>135<br>116<br>30<br>64<br>134<br>28<br>12<br>50<br>9<br>1<br>1	6.2 %<br>6.0 %<br>7.9 %<br>5.1 %<br>4.7 %<br>4.6 %<br>8.0 %<br>6.0 %<br>4.4 %<br>5.2 %<br>6.2 %<br>5.1 %<br>4.8 %<br>4.3 %<br>4.5 %<br>5.2 %	0.002
	Aragón	553				
	Asturias	447				
	Baleateares	453				
	Canarias	887				
	Cantabria	251				
	Castilla y León	1052				
	Castilla La-Mancha	863				
	Cataluña	2934				
	Comunidad Valenciana	2118				
	Extremadura	451				
	Galicia	1190				
	Madrid	2670				
	Murcia	622				
	Navarra	266				
	País Vasco	911				
	La Rioja	132				

					6.2 % 3.4% 4.0%	
	Ceuta	29				
	Melilla	30				

Breakfast	At home	16243	94.0 %	1045	6.0 %	**<0.001**
	Away from home	2287	98.0 %	47	2.0 %	
	Don't usually have breakfast	760	98.1 %	15	1.9 %	
Fruit consumption	Daily	11655	93.8 %	772	6.2 %	**<0.001**
	> 3 times/week	3774	95.4 %	182	4.6 %	
	once-twice/week	2185	96.1 %	88	3.9 %	
	< once/week	761	96.8 %	25	3.2 %	
	Never or hardly ever	914	95.9 %	39	4.1 %	
Vegetables consumption	Daily	9053	94.6 %	513	5.4 %	0.427
	> 3 times/week	6595	94.3 %	395	5.7 %	
	once-twice/week	2622	95.0 %	139	5.0 %	
	< once/week	657	95.2 %	33	4.8 %	
	Never or hardly ever	363	93.0 %	27	7.0 %	
Legume consumption	Daily	298	95.5 %	14	4.5 %	0.046
	> 3 times/week	4449	93.9 %	289	6.1 %	
	once-twice/week	11515	94.7 %	646	5.3 %	

	< once/week	2290	95.4 %	109	4.6 %	
	Never or hardly ever	737	93.8 %	49	6.2 %	
Dairy consumption	Daily	16281	94.5 %	950	5.5 %	0.019
	> 3 times/week	1487	95.5 %	70	4.5 %	
	once-twice/week	677	96.3 %	26	3.7 %	
	< once/week	328	94.2 %	20	5.8 %	
Sweet consumption	Never or hardly ever	517	92.5 %	42	7.5 %	
	Daily	5587	94.3 %	337	5.7 %	<0.001
	> 3 times/week	2977	95.9 %	127	4.1 %	
	once-twice/week	3532	95.3 %	176	4.7 %	
	< once/week	3134	95.6 %	144	4.4 %	
	Never or hardly ever	4060	92.6 %	322	7.4 %	
Fast food	Daily	358	99.6 %	1	0.4 %	**<0.001**
	> 3 times/week	904	98.4 %	14	1.6 %	
	once-twice/week	4017	98.5 %	61	1.5 %	
	< once/week	4764	98.0 %	97	2.0 %	
	Never or hardly ever	9246	90.8 %	933	9.2 %	
Dental hygiene	3 or more times/day	7523	97.0 %	236	3.0 %	**<0.001**
	> twice/day	6449	95.7 %	289	4.3 %	
	once/day	3731	92.5 %	304	7.5 %	
	Never or occasionally	1587	85.1 %	279	14.9 %	

Self-perceived health	Very good	4353	99.0 %	43	1.0 %	**<0.001**
	Good	10116	97.1 %	297	2.9 %	
	Fair	3651	89.8 %	414	10.2 %	
	Bad	966	78.9 %	258	21.1 %	
	Very bad	204	68.4 %	94	31.6 %	
Any chronic disease	No	11578	97.9 %	245	2.1 %	**<0.001**
	Yes	7712	89.9 %	862	10.1 %	
AH	No	15566	96.6 %	553	3.4 %	**<0.001**
	Yes	3724	87.0 %	554	13.0 %	
AMI	No	19007	95.1 %	990	4.9 %	**<0.001**
	Yes	283	70.7 %	117	29.3 %	
Other heart disease	No	18382	95.6 %	845	4.4 %	**<0.001**
	Yes	908	77.6 %	262	22.4 %	
Varicose veins	No	17200	95.1 %	887	4.9 %	**<0.001**
	Yes	2090	90.5 %	220	9.5 %	
Osteoarthritis	No	16101	96.6 %	562	3.4 %	**<0.001**
	Yes	3189	85.4 %	545	14.6 %	
Chronic neck pain	No	16252	95.3 %	801	4.7 %	**<0.001**
	Yes	3038	90.9 %	306	9.1 %	
Chronic low back pain	No	15682	95.5 %	746	4.5 %	**<0.001**
	Yes	3608	90.9 %	361	9.1 %	

Allergy	No	16899	94.3 %	1022	5.7 %	**<0.001**
	Yes	2391	96.6 %	85	3.4 %	
Asthma	No	18337	94.7 %	1022	5.3 %	**<0.001**
	Yes	953	91.8 %	85	8.2 %	
COPD	No	18552	95.3 %	907	4.7 %	**<0.001**
	Yes	738	78.7 %	200	21.3 %	
Diabetes mellitus	No	18092	95.6 %	841	4.4 %	**<0.001**
	Yes	1198	81.8 %	266	18.2 %	
Stomach ulcer	No	18534	94.8 %	1011	5.2 %	**<0.001**
	Yes	756	88.7 %	96	11.3 %	
Urinary incontinence	No	18815	95.5 %	895	4.5 %	**<0.001**
	Yes	475	69.1 %	212	30.9 %	
High cholesterol	No	15828	95.3 %	789	4.7 %	**<0.001**
	Yes	3462	91.6 %	318	8.4 %	
Cataracts	No	18067	96.2 %	712	3.8 %	**<0.001**
	Yes	1223	75.6 %	395	24.4 %	
Skin problems	No	18408	94.7 %	1024	5.3 %	**<0.001**
	Yes	882	91.4 %	83	8.6 %	
Chronic constipation	No	18588	95.0 %	984	5.0 %	**<0.001**
	Yes	702	85.1 %	123	14.9 %	
Cirrhosis	No	19126	94.7 %	1068	5.3 %	**<0.001**

	Yes	163	80.8 %	39	19.2 %	
Depression	No	17986	95.1 %	932	4.9 %	**<0.001**
	Yes	1304	88.2 %	175	11.8 %	
Anxiety	No	17858	94.8 %	977	5.2 %	**<0.001**
	Yes	1431	91.7 %	130	8.3 %	
Other mental problems	No	19098	95.0 %	1011	5.0 %	**<0.001**
	Yes	192	66.5 %	97	33.5 %	
Ictus	No	19133	94.9 %	1030	5.1 %	**<0.001**
	Yes	157	67.0 %	77	33.0 %	
Migraine	No	17413	94.5 %	1006	5.5 %	**0.507**
	Yes	1877	94.9 %	101	5.1 %	
Haemorrhoids	No	18199	94.8 %	1001	5.2 %	**<0.001**
	Yes	1091	91.2 %	106	8.8 %	
Malignant tumours	No	18852	95.1 %	969	4.9 %	**<0.001**
	Yes	438	76.1 %	138	23.9 %	
Osteoporosis	No	18606	95.1 %	964	4.9 %	**<0.001**
	Yes	684	82.7 %	143	17.3 %	
Thyroid problems	No	18281	94.6 %	1043	5.4 %	0.425
	Yes	1009	94.1 %	64	5.9 %	
Permanent injuries due to accidents	No	18265	94.6 %	1035	5.4 %	0.088
	Yes	1025	93.4 %	72	6.6 %	

Accidents<br><br>(last year)	No	17700	94.7 %	999	5.3 %	0.076
	Yes	1590	93.6 %	108	6.4 %	
GHQ12 mental health	Absence of psychopathology	17007	95.6 %	788	4.4 %	**<0.001**
	Suspicion	930	93.6 %	64	6.4 %	
	Presence of psychopathology	1184	86.6 %	183	13.4 %	
	NR/DK	169	70.3 %	71	29.7 %	
Activity limitation<br><br>(last 2 weeks)	No	17266	95.5 %	811	4.5 %	**<0.001**
	Yes	2024	87.2 %	296	12.8 %	
Bed rest	No	18346	95.1 %	951	4.9 %	**<0.001**
(last 2 weeks)	Yes	944	85.8 %	156	14.2 %	
Activity limitation	Severely limited	430	64.0 %	242	36.0 %	**<0.001**
(last 6 months)	Non-severe limited	2866	88.5 %	372	11.5 %	
	Not limited	15994	97.0 %	493	3.0 %	
Use of glasses or contact lenses	No	7721	97.1 %	232	2.9 %	**<0.001**
	Yes	11569	93.0 %	875	7.0 %	
Use of hearing aid	No	18763	94.9 %	1000	5.1 %	**<0.001**
	Yes	527	83.1 %	107	16.9 %	
Hospital admission	No	17855	95.5 %	847	4.5 %	**<0.001**
(last year)	Yes	1435	84.6 %	260	15.4 %	
General practitioner visit	No	14085	96.0 %	593	4.0 %	**<0.001**
(last month)	Yes	5205	91.0 %	514	9.0 %	

Specialist visit	No	16497	95.2 %	834	4.8 %	**<0.001**
(last month)	Yes	2793	91.1 %	273	8.9 %	
Day hospital (last year)	No	17901	94.9 %	957	5.1 %	**<0.001**
	Yes	1389	90.3 %	150	9.7 %	
Emergency visits (last year)	No	14236	95.4 %	690	4.6 %	**<0.001**
	Yes	5054	92.4 %	417	7.6 %	
Psychologist visit (last year)	No	18634	94.6 %	1066	5.4 %	**<0.001**
	Yes	656	94.1 %	41	5.9 %	
X-ray (last year)	No	14173	95.6 %	658	4.4 %	**<0.001**
	Yes	5117	91.9 %	449	8.1 %	
CT Scan	No	17976	95.3 %	885	4.7 %	**<0.001**
(last year)	Yes	1314	85.5 %	222	14.5 %	
Ultrasound (last year)	No	16594	95.0 %	878	5.0 %	**<0.001**
	Yes	2696	92.2 %	229	7.8 %	
MRI (last year)	No	17926	94.9 %	958	5.1 %	**<0.001**
	Yes	1364	90.1 %	149	9.9 %	
Influenza vaccine (last campaign)	No	16123	97.1 %	487	2.9 %	**<0.001**
	Yes	3167	83.6 %	620	16.4 %	

BMI: Body Mass Index; VT: Vocational Training; AH: Arterial hypertension; AMI: Acute Myocardial Infarction; COPD: Chronic Obstructive Pulmonary Disease; GHQ: General Health Questionnaire; CT scan: Computed Axial Tomography; MRI: Magnetic Resonance Imaging.

Table S4. Relative Risks estimated by Poisson models with robust variance.

		RR	CI 95%	p-value
Physiotherapist visit (last year)	Yes	0.699	(0.528-0.927)	0.013
BMI	Normal			
	Overweight	0.832	(0.699-0.992)	0.040
	Obesity	0.936	(0.77-1.137)	0.504
	NR/DK	1.081	(0.905-1.291)	0.391
Marital status	Single			
	Married	0.617	(0.488-0.779)	**<0.001**
	Widowed	0.758	(0.602-0.955)	0.019
	Separated	0.770	(0.505-1.174)	0.225
	Divorced	0.703	(0.389-1.271)	0.243
Net monthly household income	NR/DK			
	> € 2251	1.081	(0.792-1.475)	0.624
	€ 1551-2250	0.875	(0.69-1.11)	0.272
	€ 1051-1550	0.955	(0.779-1.171)	0.657
	€ 801-1050	0.915	(0.74-1.132)	0.415
	< € 800	0.935	(0.789-1.109)	0.441
Tobacco consumption	Never			
	Ex-smoker	1.085	(0.903-1.305)	0.384
	Smoker	1.479	(1.161-1.883)	0.002
Main activity daily	Sitting most of the time			
	Standing most of the time	0.659	(0.544-0.799)	**<0.001**
	Walking with weight	0.756	(0.521-1.097)	0.141
	Strenuous tasks	0.607	(0.201-1.834)	0.376
Leisure physical activity	Sedentary			
	Occasional physical activity	0.738	(0.619-0.88)	0.001
	Frequent physical activity	0.592	(0.337-1.039)	0.068
	Sports training	0.631	(0.391-1.018)	0.059

Legume consumption	Daily			
	> 3 times/week	0.998	(0.589-1.691)	0.995
	once-twice/week	0.968	(0.577-1.624)	0.902
	< once/week	0.958	(0.549-1.672)	0.881
		RR	CI 95%	p-value
	Never or hardly ever	1.395	(0.789-2.468)	0.253
Dental Hygiene	3 or more times/day			
	twice/day	1.326	(1.092-1.61)	0.004
	once/day	1.140	(0.948-1.372)	0.164
	Never or occasionally	1.275	(1.054-1.543)	0.012
Self-perceived health	Very good			
	Good	1.437	(0.949-2.175)	0.086
	Fair	1.994	(1.31-3.035)	0.001
	Bad	2.307	(1.494-3.563)	**<0.001**
	Very bad	2.609	(1.65-4.125)	**<0.001**
SEX	Woman	0.605	(0.504-0.726)	**<0.001**
AGE	(years old)	1.080	(1.072-1.088)	**<0.001**
Any chronic disease	Yes	1.024	(0.862-1.217)	0.784
AMI	Yes	1.446	(1.158-1.805)	0.001
Allergy	Yes	0.749	(0.594-0.945)	0.015
COPD	Yes	1.297	(1.099-1.531)	0.002
DM	Yes	1.294	(1.118-1.497)	0.001
High cholesterol	Yes	0.751	(0.65-0.867)	<0.001
Depression	Yes	1.181	(0.977-1.427)	0.085
Malignant tumours	Yes	1.437	(1.16-1.778)	0.001
Osteoporosis	Yes	1.075	(0.89-1.298)	0.456
Activity limitation (last 2 weeks)	Yes	1.088	(0.93-1.272)	0.294
Hospital admission (last year)	Yes	1.075	(0.905-1.277)	0.410
General practitioner visit (last month)	Yes	0.939	(0.827-1.066)	0.333
Specialist visit (last month)	Yes	1.039	(0.89-1.214)	0.625
Day hospital visit (last year)	Yes	1.066	(0.888-1.281)	0.493
CT scan (last year)	Yes	1.103	(0.896-1.356)	0.355

Ultrasound (last year)	Yes	1.173	(0.956-1.439)	0.126
MRI (last year)	Yes	1.161	(0.933-1.446)	0.181

BMI: Body Mass Index; AMI: Acute Myocardial Infarction; COPD: Chronic Obstructive Pulmonary Disease; DM: Diabetes Mellitus; CT scan: Computed Axial Tomography scan. n train = 14191; nº deaths train= 1040; LRT=2102.2 ($p<0.001$); n test = 6206; nº deaths test = 439; ROC test area= 0.921; 95% CI= (0.908-0.933)
